# The Effect of Salinity on the Dielectric Permittivity of Nanoconfined Geofluids

**DOI:** 10.1021/acsearthspacechem.4c00210

**Published:** 2024-11-05

**Authors:** Alireza Chogani, Helen E. King, Aleksandar Živković, Oliver Plümper

**Affiliations:** Department of Earth Sciences, Utrecht University, Utrecht 3584 CB, The Netherlands

**Keywords:** salt concentration, nanoconfinement, water permittivity, nanoporosity, molecular dynamics

## Abstract

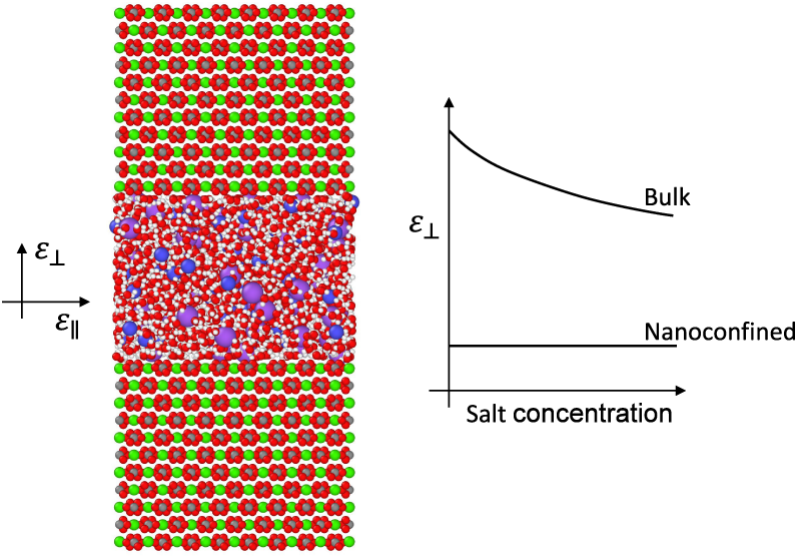

Nanoporosity is a characteristic feature of geological formations that provides potential pathways for geofluids to meander and interact with minerals. Confinement of water within nanopores leads to unique phenomena. The dielectric constant of water becomes anisotropic and adopts tensorial properties rather than remaining a scalar value. In such nanoconfinement, it has been found that the permittivity of water decreases perpendicularly and increases parallel to the interface. As geofluids are rarely pure water in nature, being a water–salt(−gas) mixture within the Earth, it becomes pivotal to examine how these additional constituents of water affect the permittivity of fluids confined within the nanopores of rocks. In this study, we present the calculation of the permittivity of saline water in calcite slit nanopores using molecular dynamics simulations under low-pressure–temperature conditions. The dielectric properties are weakly dependent on salinity for both the perpendicular and parallel dielectric permittivity components. We analyzed the atomic charge and polarization density of the fluid perpendicular to the nanochannel walls and the orientation of water molecules’ dipole inside the nanochannel. From our analysis, most of these factors were generally not altered significantly in the presence of salinity. These findings are significant because they enable us to use well-studied pure water properties under nanoconfinement to determine the geochemical behavior of fluids within natural nanoporous systems.

## 1 Introduction

The interplay between aqueous (water-based) fluids and geological formations plays a crucial role in many Earth-related phenomena, influencing different processes from the dynamics of the planet’s surface climate to activities within the Earth.^1−6^ This interaction is also key for several human activities, including the extraction and storage of natural energy resources^7^ and carbon dioxide^8^ as well as the creation of critical minerals necessary for sustainable energy technologies.^9^ Therefore, understanding the porosity and permeability of the Earth is critical for exploring how fluids move and chemically interact within different geological environments. The configuration of pores and fractures influences fluid dynamics and chemical reactions within rocks, governed by factors such as pore size and interconnectivity.^10−12^ These factors dictate the accessibility of mineral surfaces to fluids and modulate the dominance of either advective or diffusive transport mechanisms.^13^ At the microscopic scale, the intricate interactions between fluids and mineral surfaces at pore interfaces and grain boundaries affect fluid mobility and reaction processes.^14−16^ This complexity is paramount for comprehending phenomena such as multiphase flow and reactive transport, albeit their investigation is impeded by the diminutive scale of these features. Specifically, nanopores, which are pores smaller than 100 nm, are integral in this context. Ubiquitous across nearly the entire spectrum of geological materials, they substantially influence overall porosity and the mechanisms by which aqueous fluids navigate through these substrates.^16−20^

Within the confined spaces of nanopores, water exhibits distinct properties from its bulk state, including enhanced flow rates,^21,22^ altered density,^23^ phase changes,^24,25^ and variations in its capacity for electric charge storage and transfer, known as relative permittivity.^26−30^ Relative permittivity is a measure of a material’s ability to store and transmit electric energy in an electric field, influencing how the material polarizes in response to the field. This concept is pivotal as it influences electrostatic interactions between charged particles, thereby controlling chemical reactions, as well as the solvation and mobility of ions within the solution. Water has a high relative permittivity, which makes it particularly adept at dissolving ionic compounds, facilitating the creation of ionic solutions. However, a marked decrease in water’s relative permittivity is found within nanopore environments, signifying a substantial shift in its electrical and chemical properties in confined conditions.^26,27,30^ Water in the Earth’s crust frequently contains dissolved electrolytes, predominantly NaCl and/or CaCl_2_, potentially with K, Mg, and Fe salts under specific conditions.^31^ Interestingly, the electrolyte concentration in crustal fluids is not solely determined by the solubility of chloride mineral salts but can vary widely, often surpassing typical surface conditions. This variability underscores the need to explore how the presence of “salts” affects the permittivity of water in nanoporous structures, potentially altering its electrical properties and hence influencing fluid–rock interaction.

Research on the dielectric responses of electrolytes confined in cylindrical silica nanopores and graphene nanochannels has revealed variations in the relative permittivity with salt concentration, potentially stemming from intricate interactions between ions and the hydrogen-bond network of water molecules, as well as changes in the orientation of water’s dipole moments.^32−38^ Despite these advancements, the dynamics of fluids within nanoporous geological structures remain largely unknown. Here, we use molecular dynamics simulations to investigate the dielectric permittivity of saline water with varying NaCl concentrations confined between nanospaced calcite surfaces. We compute both perpendicular and parallel dielectric permittivity components. We quantify the influence of salt ions, focusing on atomic charge density, polarization, and water molecules’ dipole orientation variations. Our findings contribute to a quantitative understanding of the electrical properties of nanoconfined geofluids. In the following, we explain the simulation methods and permittivity calculations in detail, accompanied by an in-depth presentation of the results and discussion.

## 2 Methods

### 2.1 Molecular Dynamics (MD) Simulation

Calcite surfaces were obtained using density functional theory (DFT) calculations for subsequent MD simulations. We selected calcite because it is a prevalent mineral in environments close to the Earth’s surface and is especially significant in carbon dioxide storage schemes.^39−42^ Detailed information on the DFT methods is provided in the Supporting Information. For MD simulations, we utilized the LAMMPS package.^43^ The integration of equations of motion was performed using the velocity-Verlet algorithm, with a time step of 1 femtosecond (fs). To maintain the temperature constant at 300 K throughout the simulations, a Nosé–Hoover thermostat was applied with a time constant of 0.1 picoseconds (ps).^44^ Following an equilibration period of 2 nanoseconds (ns), simulations were conducted in a canonical ensemble (*NVT*) until the permittivity fully converged (20 up to 70 ns duration). The cutoff radius for short-range interactions was established at 1 nanometer (nm) for Lennard-Jones (LJ) potentials and 1.2 nm for Coulomb potentials. Long-range electrostatic interactions were calculated using the particle–particle particle-mesh (PPPM) solver.^45^ Periodic boundary conditions were enforced in the *x* and *y* directions, with the Yeh–Berkowitz slab correction method applied to mitigate the influence of long-ranged electrostatic interactions from periodic image cells in the *z*-direction.^46^ The Yeh–Berkowitz correction modifies the standard Ewald summation technique to account for systems with slab geometry, where the system is periodic in two dimensions (*x* and *y*) but finite in the third dimension (*z*). The Yeh–Berkowitz correction introduces an additional term to the Ewald summation to correct errors caused by dipole moment interactions from periodic image cells in the *z*-axis. This correction enhances both the accuracy and efficiency of simulations for systems such as interfaces or confined fluids. Atom trajectories were recorded every 0.1 ps, providing a substantial data set for permittivity calculations.

The simulated confined systems comprised saline water situated between two parallel calcite slabs, separated by distances ranging from 1 to 3 nm in the *z*-direction. The slabs were kept static throughout the simulation. This rigidity helps to prevent artifacts that could result from unrealistic conformational changes in the substrate. Additionally, our findings show that the first water layer is located 2.33 Å from the calcium carbonate surface, while the second layer is at 3.63 Å. This arrangement of interfacial water is consistent with both experimental data and simulation studies on calcite–water interfaces, such as those discussed by Le et al.^47^ Moreover, calculating the perpendicular permittivity involves integrating atomic charge and polarization density profiles along the *z*-axis throughout the simulation. The rigidity of the substrate provides a fixed reference plane (*z* = 0) for this integration, in line with common approaches used in dielectric calculations under confined conditions (e.g., refs (27) and (30)). Water molecules were modeled using the extended simple point charge (SPC/E) model,^48^ with the SHAKE algorithm^49^ employed to maintain their structural rigidity. The SPC/E model includes a polarization correction to the effective pair potential, which enhances its ability to predict the dielectric properties of water. This enables SPC/E to more accurately estimate the dielectric constant of water, with a value of 71 at room temperature, surpassing other commonly used water models such as TIP3P and TIP4P.^27,50^ Additionally, SPC/E has demonstrated reliability under extreme pressure–temperature conditions, making it well-suited for investigating geological processes.^51,52^ The results were averaged over five MD simulations with different initial conditions to ensure accuracy. The number of water molecules for each system was adjusted through an insertion/deletion process to match the bulk water density under ambient conditions.^30^ By doing so, we indirectly maintained atmospheric pressure in the simulations, as the temperature was controlled using the applied thermostat. The calcite surface was modeled based on the (10–14) plane with dimensions of 4.1 × 4 nm in the *x*–*y* plane and a thickness of 3.2 nm. The width of the pore channel was defined by the distance between the outermost oxygen atoms on the bottom and top surfaces. The (10–14) plane is the main cleavage plane, thermodynamically stable and commonly exposed surface in calcite. It plays a key role in calcite’s growth and dissolution processes, making it essential for determining the mineral’s behavior.^53,54^

Interactions between calcite and water were modeled using the force field developed by Xiao et al.^55^ Although the TIP3P water model was used in the development of this force field, Le et al.^47^ demonstrated that the structure of SPC/E water on the calcite model provided by Xiao et al. is nearly identical to that obtained using the TIP3P water model. The agreement of our interfacial water structure with previously reported findings, as mentioned earlier, further reinforces the reliability of the simulation setup used in this study. Sodium chloride (NaCl) was incorporated at concentrations varying from 0 (pure water) to 2 M, with the ion concentration within the nanochannels determined using the same approach as in the bulk solution. This salt concentration range encompasses a broad spectrum of pore fluid salinities in different crustal settings.^31^ Ion interactions with other atoms and ions were modeled using LJ and Coulomb potentials, with potential parameters adopted from Jalali et al.^35^

### 2.2 Perpendicular Permittivity Calculation

In confined environments, the dielectric permittivity of water exhibits tensorial characteristics, diverging from its behavior in bulk form. Specifically, within a slit channel there are two components of permittivity: one perpendicular to the channel wall (ε_⊥_) and another parallel to the channel wall (ε_∥_). Both permittivity components demonstrate spatial variation as a function of *z* (distance from the bottom surface). Drawing upon principles of statistical mechanics and linear response theory,^56,57^ the fluctuation equation below delineates the local inverse perpendicular permittivity:
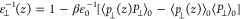
1where β is thermal energy inverse, ⟨···⟩_0_ represents the ensemble average when there is no external electric field, *p*_⊥_(*z*) is the perpendicular fluid polarization density at position *z*, and *P*_⊥_ is the fluid total polarization’s perpendicular component, which is provided by
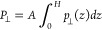
2

The perpendicular polarization density at position *z* is determined as
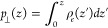
3where ρ_e_(*z*) is the fluid atomic charge density profile in the *z*-direction calculated using a binning method with 0.01 Å resolution.

When the above equation is integrated over the entire channel, the average inverse perpendicular permittivity is obtained as
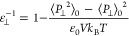
4

Originally, Ballenegger and Hansen (BH) formulated equations for the dielectric permittivity of a single slab, assuming periodicity only in the *x* and *y* dimensions.^56^ For systems that are nonuniform in the *z* direction yet exhibit periodicity in all three dimensions, the accurate fluctuation equation for determining perpendicular dielectric permittivity must incorporate fluctuations in the total polarization of the system, as described by the Stern and Feller (SF) formula.^58^ In our MD simulations, the system is designed to be periodic in the *x* and *y* directions, employing the particle–particle particle-mesh (pppm) solver to calculate long-ranged Coulomb electrostatic interactions. For the *z*-direction, however, we intentionally omit the long-range electrostatic contributions from the periodic image cells, which are considered inappropriate for two-dimensional periodic (2DP) systems. Yeh and Berkowitz demonstrated that such an approach in the *z*-direction (Ewald3dc) mirrors the accurate behavior of two-dimensional Ewald summation specific to a 2DP system.^46^ Consequently, by excluding image cell contributions in the *z*-direction and ensuring ample empty space along the same axis, we apply the BH formula to compute the perpendicular dielectric permittivity under these specified conditions. Selecting suitable boundary conditions and fluctuation equations is important to achieve reliable outcomes. Utilizing the BH equation with Ewald3d or the SF formula with Ewald3dc leads to inaccuracies, particularly at the channel’s midpoint. Validating the bulk dielectric constant value at the channel center, distant from any interfaces, acts as a critical benchmark confirming the precision of the selected permittivity equations and boundary conditions.^27^

### 2.3 Parallel Permittivity Calculation

For systems that are inhomogeneous only in one direction (the *z*-axis, perpendicular to the surface), the locally varying parallel dielectric permittivity can be calculated using principles from statistical mechanics and linear response theory with the following equation:
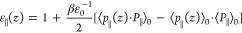
5where the vector *p*_∥_ = (*p*_*x*_,*p*_*y*_) represents the parallel polarization density of the fluid at position *z*, and *P*_∥_ denotes the parallel component of the total fluid polarization vector. These vectors can be calculated using the molecular dipole moment with the below equations:
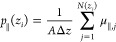
6
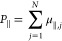
7where  represents the parallel component of the *j*th dipole, *N* denotes the total number of water molecules, *N*(*z*_*i*_) signifies the number of molecules positioned at *z* = *z*_*i*_ inside the channel, and *z*_*i*_ indicates the location of the ith bin with thickness Δ*z*. Since the system is homogeneous in the *x* and *y* directions, one can neglect the ⟨*p*_∥_(*z*)⟩_0_.⟨*P*_∥_⟩_0_ term in eq 5. Thus, by applying the definitions provided in eqs 6 and 7, we can find the parallel dielectric permittivity as below:
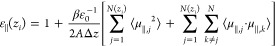
8

In this equation, the first summation pertains to the dipole–dipole self-correlation within a bin, and the subsequent one refers to the cross-correlation between the dipoles of the *i*th bin and the entire system.^29^

## 3 Results and Discussion

**Figure 1:**
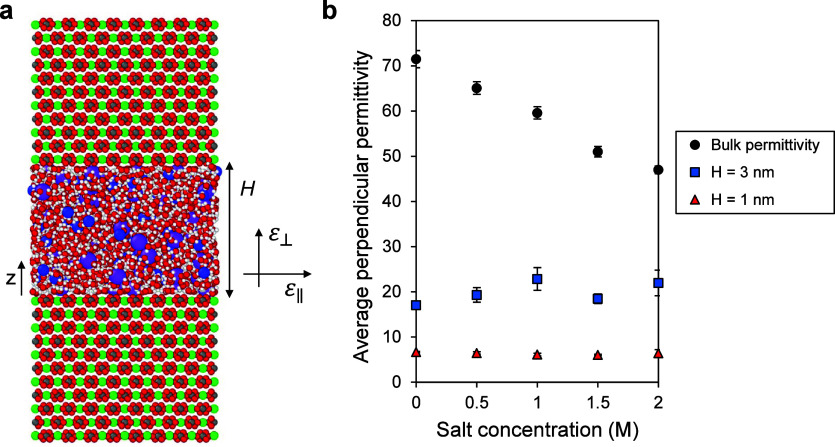
Saline water in nanoconfinement. (a) A snapshot of the simulation setup illustrating water molecules, Na^+^ and Cl^–^ ions (*c* = 2 M), between two calcite slabs separated by a distance of 3 nm. Atom colors: O—red, H—white, Na^+^—purple, Cl^–^—blue, Ca—green, C—dark gray. (b) Average perpendicular permittivity within channels of varying heights (*H* = 1 and 3 nm) as a function of NaCl concentration. Nanoconfined results are compared with the dielectric permittivity of bulk saline water obtained from MD simulations. The bulk water permittivity data points are adopted from Seal et al.^60^

Figure 1a showcases a snapshot within the simulation box, capturing water with Na and Cl ions constrained between two calcite slabs. The salt concentration (*c*) investigated ranges from 0 (pure water) to 2 M, corresponding to a broad spectrum of salinity found in crustal fluids. Based on data from drilling or preserved in fluid inclusions, sediments and metasediments deposited in oceanic or accretionary prism settings can contain fluids with a wide range of salinities, commonly below ∼1 M (nearly twice seawater salinity). Compared to oceanic environments, continental margin fluids generally have higher salinities. In the case of continental settings, with increasing metamorphic grade, an extensive range of salinities may develop, with the uppermost levels approaching halite saturation.^59^ Thus, the selected salinity range adequately encompasses the full range of oceanic fluids and a diverse range of continental fluids. We employed eq 4 to compute the average perpendicular permittivity of the saline water confined within the nanochannels. The findings are depicted in Figure 1b, illustrating the average ε_⊥_ as a function of salt concentration across two channels of heights *H* = 1 nm and *H* = 3 nm. For comparison with bulk behavior, the permittivity data of saline water calculated using molecular dynamics with the SPC/E water model adopted from Seal et al.^60^ is juxtaposed. As observed, the average ε_⊥_ remains constant in both channels as the salt concentration increases. In the case of pure water, prior research has indicated a decrease in ε_⊥_ under nanoconfinement.^26,27,30^Figure 1b shows that the average ε_⊥_ of pure water within 1 and 3 nm calcite channels registers at 6.7 and 17, respectively, markedly below the dielectric constant of bulk water, which stands at 71 under analogous temperature and pressure conditions. For bulk saline water, the static dielectric constant decreases with increasing salt concentration, a phenomenon known as dielectric decrement.^61^ This reduction is attributed to the local electric fields generated by the ions, which disrupt the external electric field’s influence. Polar water molecules arrange into a hydration shell around each ion, aligning with the local ionic field and diminishing their responsiveness to the external field, thereby reducing the dielectric constant. According to Figure 1b, it seems that nanoconfinement effects dominate over the inclusion of salt ions, as in all cases, the average ε_⊥_ is much lower than in bulk, and it remains insensitive to salt concentration. In general, nanoconfinement affects the fluid’s perpendicular permittivity in channels with sizes below 100 nm. Specifically, the dielectric permittivity begins to decrease in confinements around 100 nm, but the reduction becomes significant in pores smaller than 30 nm.^26,27,30^ The channel sizes investigated in the present study are well below this threshold, and we observe a strong influence of the surrounding solid on the fluid’s dielectric response.

**Figure 2:**
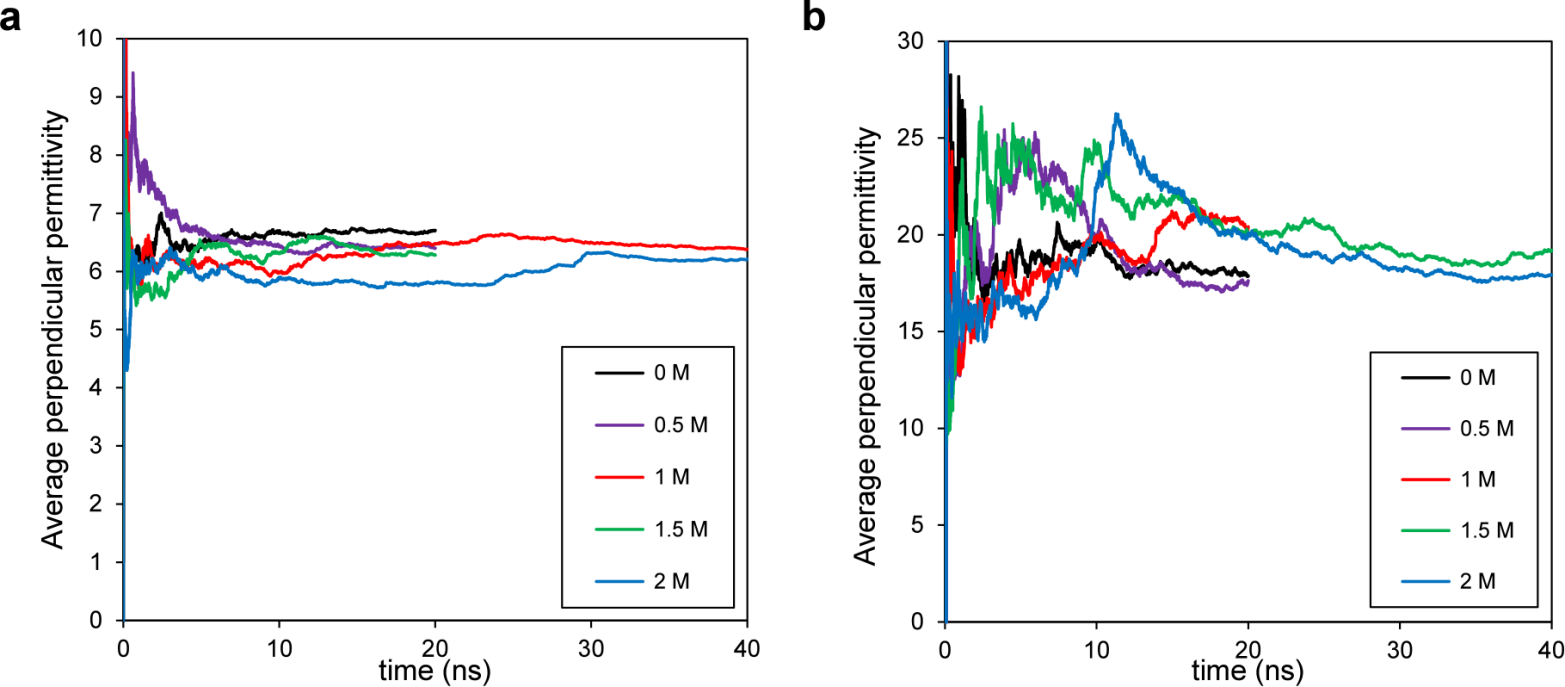
Average ε_⊥_as a function of simulation time for various channel sizes and salt concentrations. (a) *H* = 1 nm. (b) *H* = 3 nm. As salt concentration and channel size increase, more simulation time is required for average ε_⊥_ to fully converge. For a given channel size, salinity has an insignificant effect on the final average ε_⊥_ value.

Notably, we continued our simulations until we could ensure that the average ε_⊥_ throughout the entire channel had fully converged (i.e., it does not change with time anymore). Figure 2 illustrates the average ε_⊥_ as a function of simulation time for the smaller channel (*H* = 1 nm, Figure 2a) and the larger one (*H* = 3 nm, Figure 2b) with various salt concentrations. It is evident that as the salt concentration and/or system size increase, average ε_⊥_ fluctuations also increase, and it takes longer to reach a constant value. For instance, in the case of *H* = 1 nm, all simulations converged within 40 ns, whereas for *H* = 3 nm and *c* = 2 M, we needed to extend one simulation to 70 ns to ensure convergence (Figure S1). This implies that simulating larger pores requires running the simulations for longer durations (typically ranging from a few hundred nanoseconds up to a few microseconds depending on the system size and salt concentration), which is computationally expensive. Therefore, we investigated pore sizes ranging from 1 to 3 nm for the following reasons: (i) it is computationally efficient, (ii) this range covers the absence of bulk-like behaviors (*H* = 1 nm) to a combination of interfacial effects and bulk-like behavior at the pore center (*H* = 3 nm), (iii) it is prevalent in many geological structures; for instance, pores with an effective radius of 2 nm dominate the total microporosity in both soils and clays,^62^ and (iv) if there is any salinity effect on permittivity under nanoconfinement, it is expected to be more pronounced in smaller nanopores. Moreover, Figure 2 indicates that, for a given channel size, the average ε_⊥_ converges to the same value regardless of salt concentration, reaffirming our previous statement that salinity has a marginal effect on average ε_⊥_ under nanoconfinement. However, permittivity varies locally within the channel. Thus, in addition to its average value throughout the channel, the effects of salinity on its local variations must be investigated as well.

**Figure 3:**
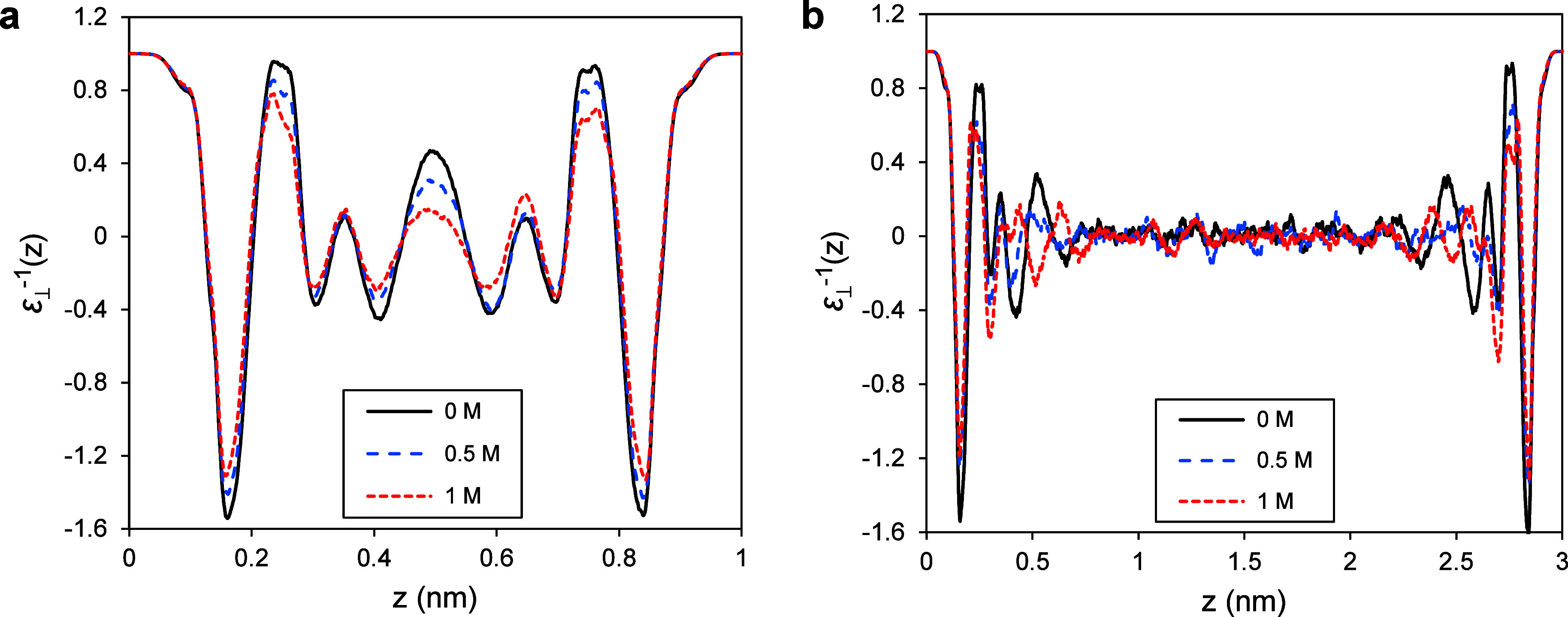
Inverse perpendicular permittivity variations inside nanochannels. (a)  of pure (*c* = 0 M) and saline water with varying salt concentrations (*c* = 0.5 and 1 M) inside a 1 nm channel. (b)  of pure and saline water (*c* = 0.5 and 1 M) inside a 3 nm channel. A bulk-like region in the center of the channel is visible, where permittivity approaches its bulk value (). In general, salinity slightly dampens  variations inside the nanochannel. The inverse permittivity representation makes small variations at the center less noticeable. The main purpose of this plot is to act as a benchmark, ensuring that the results are physically meaningful and to validate both the simulation setup and the equations applied.

To further explore the influence of salinity, Figure 3 presents the inverse perpendicular permittivity profiles across channels of different heights and salt concentrations. In the instance of the narrow channel (*H* = 1 nm, Figure 3a), no discernible bulk-like region is apparent. However, for the larger channel (*H* = 3 nm, Figure 3b), a substantial bulk-like region emerges beyond the interfacial layers, notably at the center, away from the interfaces. In these profiles, a bulk-like region is characterized by fluctuations around 0.014, as indicated by  (ref (27)). We want to emphasize that this is an important benchmark to ensure the validity of the results. Motevaselian and Aluru^27^ demonstrated in detail how an incompatible set of equations and boundary conditions can lead to physically incorrect outputs. Notably, this plot shows the inverse of permittivity, which makes subtle variations at the center less visible. However, the surrounding solid’s control on the fluid’s perpendicular permittivity extends over several nanometers, impacting the entire confined space. This effect is due to long-range dipolar correlations, known as the excluded-volume effect. Specifically, in nanoconfinement, low-dielectric solid walls replace volumes that would be filled by water in bulk, thereby excluding some long-range water dipolar interactions and contributing to the reduction in dielectric permittivity.^30^ According to Figure 3, it appears that salinity slightly dampens  fluctuations, as evidenced by lower peaks and shallower valleys observed with increasing salt concentration.

To elucidate the origin of salinity effects on perpendicular permittivity, it is crucial to recognize that, according to eq 1, perpendicular permittivity is determined by perpendicular polarization density fluctuations. This polarization density, in turn, emanates from the atomic charge density in the *z*-direction (eq 3). As each atom carries a partial charge, the spatial arrangement of all atoms within the simulation box governs the atomic charge density. This implies that the spatial distribution of ions and water molecules within the channel governs the fluid’s dielectric behavior.

**Figure 4:**
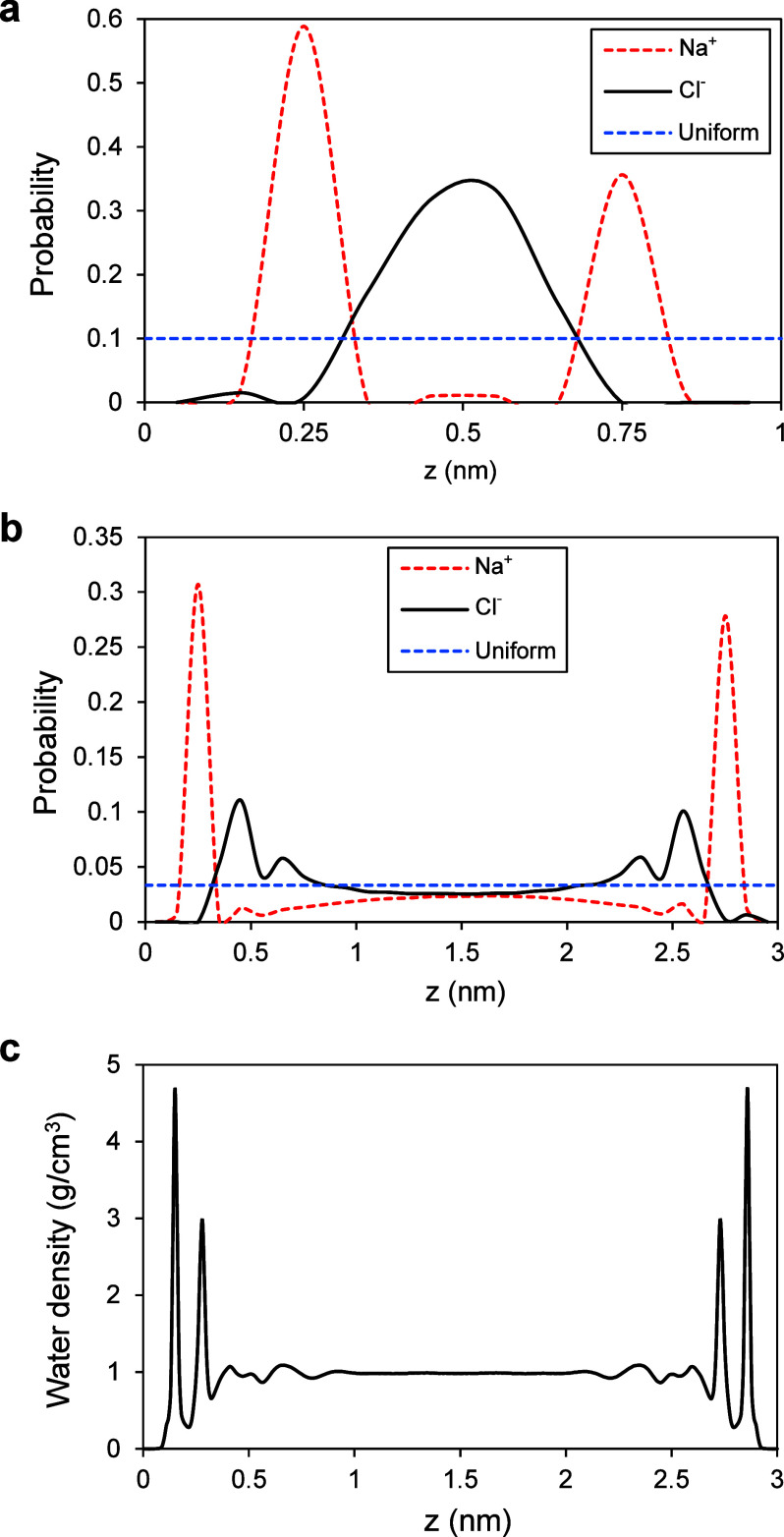
Ions and water molecules distribution inside calcite nanochannels. (a) Na^+^ and Cl^–^ spatial distribution inside a 1 nm channel. Na^+^ exhibits a peak at 0.25 nm distance from the interface, and Cl^–^ accumulates at the center. An electric double layer is formed without any bulk-like region. (b) Ions distribution inside a 3 nm channel. Ions show a radial distribution inside the channel, with a peak close to the interface and approaching homogeneous bulk conditions away from the interface. The horizontal dashed lines show uniform distributions in (a and b). (c) Water density profile inside a 3 nm channel showing two peaks close to the interface and approaching the bulk water density at ambient conditions (1 g/cm^3^) in the center.

**Figure 5:**
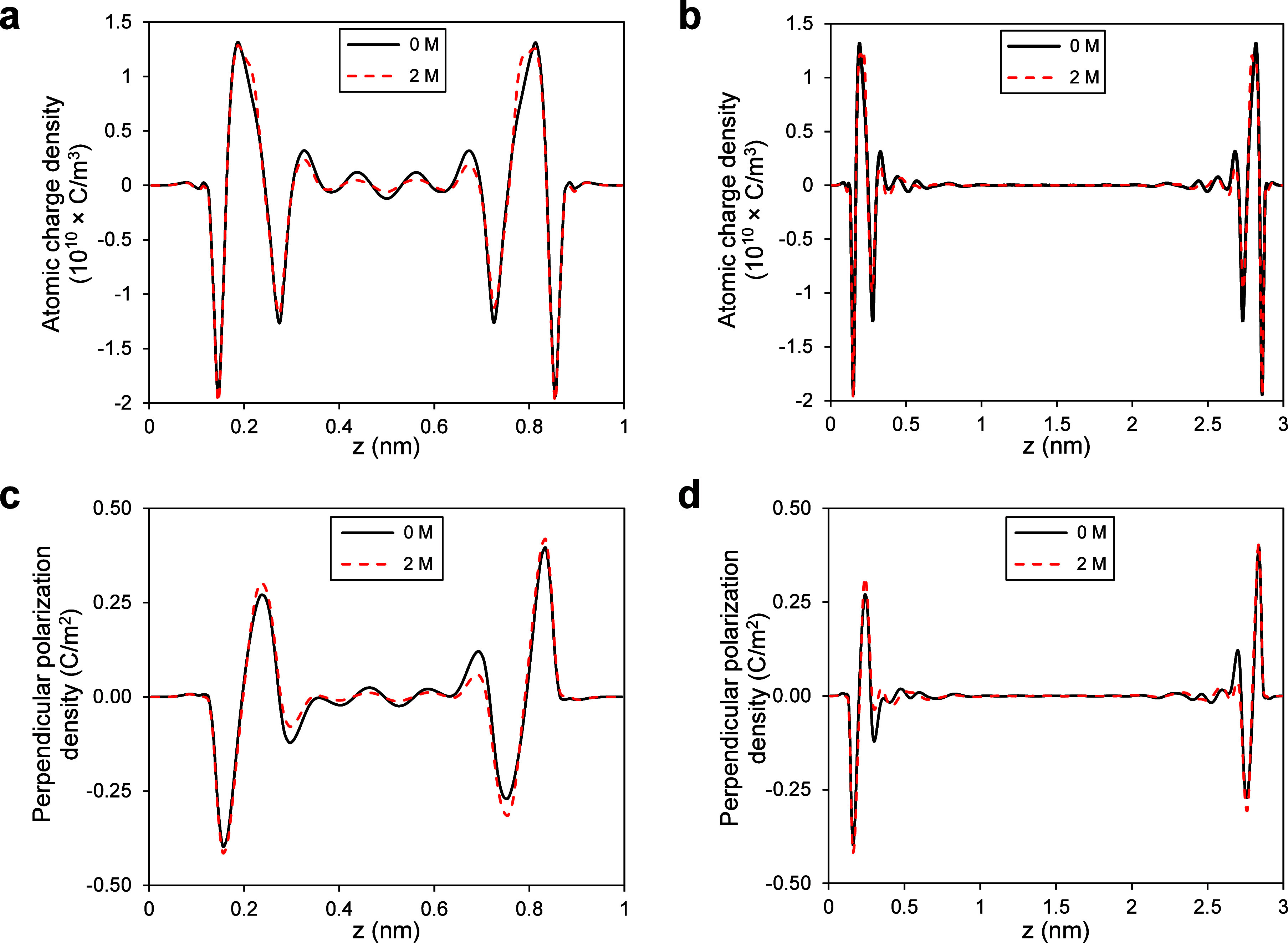
Atomic charge and perpendicular polarization density variations inside calcite nanochannels. (a, b) Atomic charge density profiles for pure and saline water (*c* = 2 M) inside 1 and 3 nm channels, respectively. (c, d) Perpendicular polarization density profiles for pure and saline water (*c* = 2 M) inside 1 and 3 nm channels, respectively. In general, all profiles are extremely similar, with the least deviations due to salinity.

Figure 4a,b depicts ion distribution profiles along the *z*-direction within channels with heights of 1 and 3 nm, respectively. A distinct double-layer formation is evident at the interfaces, characterized by sodium ions predominantly accumulating near the surface, reaching a peak around 2.5 Å, succeeded by chloride ions peaking approximately 5 Å from the surface. Additionally, ions exhibit a radial distribution concerning the channel wall, showcasing a notable peak near the interface followed by an approach toward bulk values at the channel’s central region, away from the interfaces. Figure 5c illustrates water density profiles within a channel of height *H* = 3 nm. Notably, two prominent peaks are observed in proximity to the interface. It is discernible that a dense water layer resides at the interface, succeeded by a stratum of sodium ions, another dense water layer, and finally, a layer of chloride ions. Subsequently, all concentrations gradually converge toward homogeneous bulk conditions at the channel’s central zone, distanced from the interfaces. To comprehend how ion distribution within the channel influences ε_⊥_, it becomes imperative to assess its impact on atomic charge density, which governs polarizability, and thereby, permittivity.

Figure 5a,b presents atomic-charge-density profiles for both pure and saline water (*c* = 2 M) within a 1 nm and a 3 nm calcite channels, respectively. We observe high fluctuations in the interfacial layers, indicating that the surface significantly influences the spatial distribution of water and ions. It is observed that beyond a distance of 2 Å from the surface, ions begin to influence the atomic charge density. However, even in the case of the highest investigated salt concentration (*c* = 2 M), the deviation is extremely small, and atomic charge density profiles remain nearly identical. The same behavior is observed in the perpendicular polarization density profiles as well (as shown in Figure 5c,d), where salinity has a marginal effect. This implies that the confinement effects on permittivity are much stronger than the effects of salinity, and the reorientation of water dipoles is controlled by the surface rather than salt ions.

**Figure 6:**
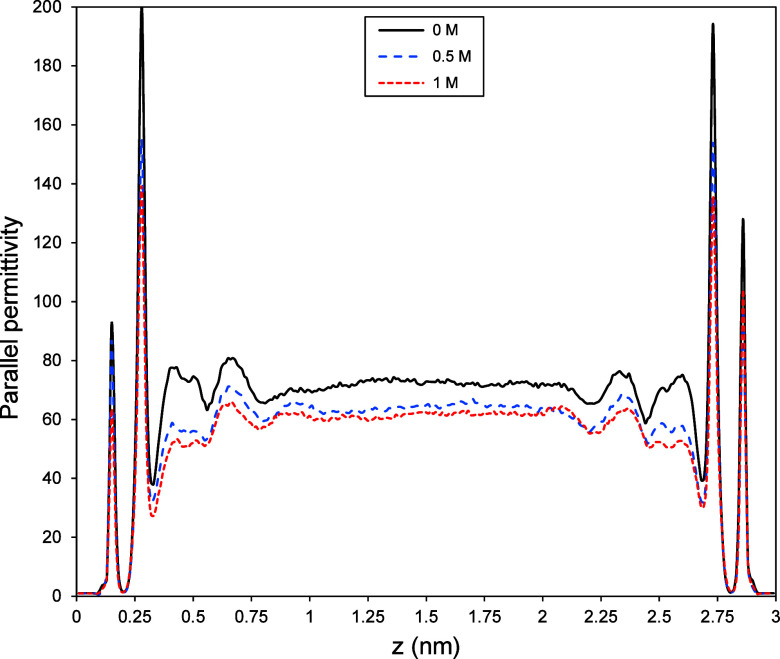
Parallel permittivity profiles of water inside a 3 nm calcite channel both in the absence and presence of salt ions in the system (*c* = 0.5 and 1 M). Notably, we observe similar behavior for all cases with peaks of ε_∥_ near the surface, which gradually approach bulk behavior in the central region. Moreover, as salinity increases, the peaks decrease slightly, accompanied by lower constant values in the center.

In addition to the perpendicular permittivity component, the parallel permittivity (ε_∥_) of water significantly deviates from its bulk counterpart under nanoconfinement. Figure 6 illustrates the variations of ε_∥_ inside a 3 nm channel for water molecules, both without any salt ions in the system and with salt ions at various concentrations (*c* = 0.5 and 1 M). We observe two peaks of ε_∥_ in the interfacial layers, after which ε_∥_ gradually approaches the bulk dielectric constant in the center of the channel. These ε_∥_ peaks near the surface correspond to peaks in water density (as shown in Figure 4c). However, high-density layers are not the sole reason for the high ε_∥_ near the surface; it also stems from water molecules’ dipolar correlations in the interfacial regions.^29^ As evident from Figure 6, in general, saline water exhibits the same behavior as pure water in terms of parallel permittivity. However, with increasing salt concentration, the peaks of ε_∥_ are slightly lower, and as expected, ε_∥_ approaches lower dielectric constant values in the center of the nanochannel for higher salt concentrations. These observed variations in ε_∥_ in the center of the channel with salinity, in contrast to ε_⊥_, could be due to the different nature and effective range of surface control on permittivity components under nanoconfinement. It appears that the surrounding solid influences ε_⊥_ over a range of several nanometers.^27,30^ However, the surface’s control over ε_∥_ is relatively short-ranged, and changes in ε_∥_ due to nanoconfinement are limited to the interfacial layers.^29^ Since ε_∥_ is derived from water molecules’ dipole–dipole correlations (see eq 8), we conducted an analysis of water molecules’ dipole orientation inside the nanochannel, both with and without salt ions in the system, to better comprehend the source of salinity effects.

**Figure 7:**
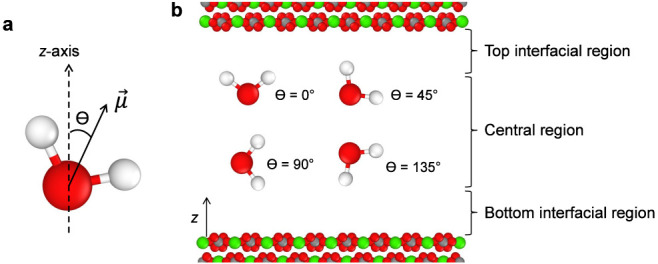
Schematic illustration of water molecules’ dipole moment orientation inside calcite nanochannels. (a) Definition of the angle (θ) between the dipole moment vector (μ) of water molecules and the *z*-axis (perpendicular to the surfaces). (b) The nanochannel is split into three distinct regions: the top interfacial, central, and bottom interfacial, as schematically illustrated. The thickness of the interfacial regions is considered to be 5 Å. Angles close to 0° correspond to the dipole moment vector being perpendicular to the calcite surface, while θ = 90° implies orientations parallel to the confining surfaces. The figure is not to scale.

**Figure 8:**
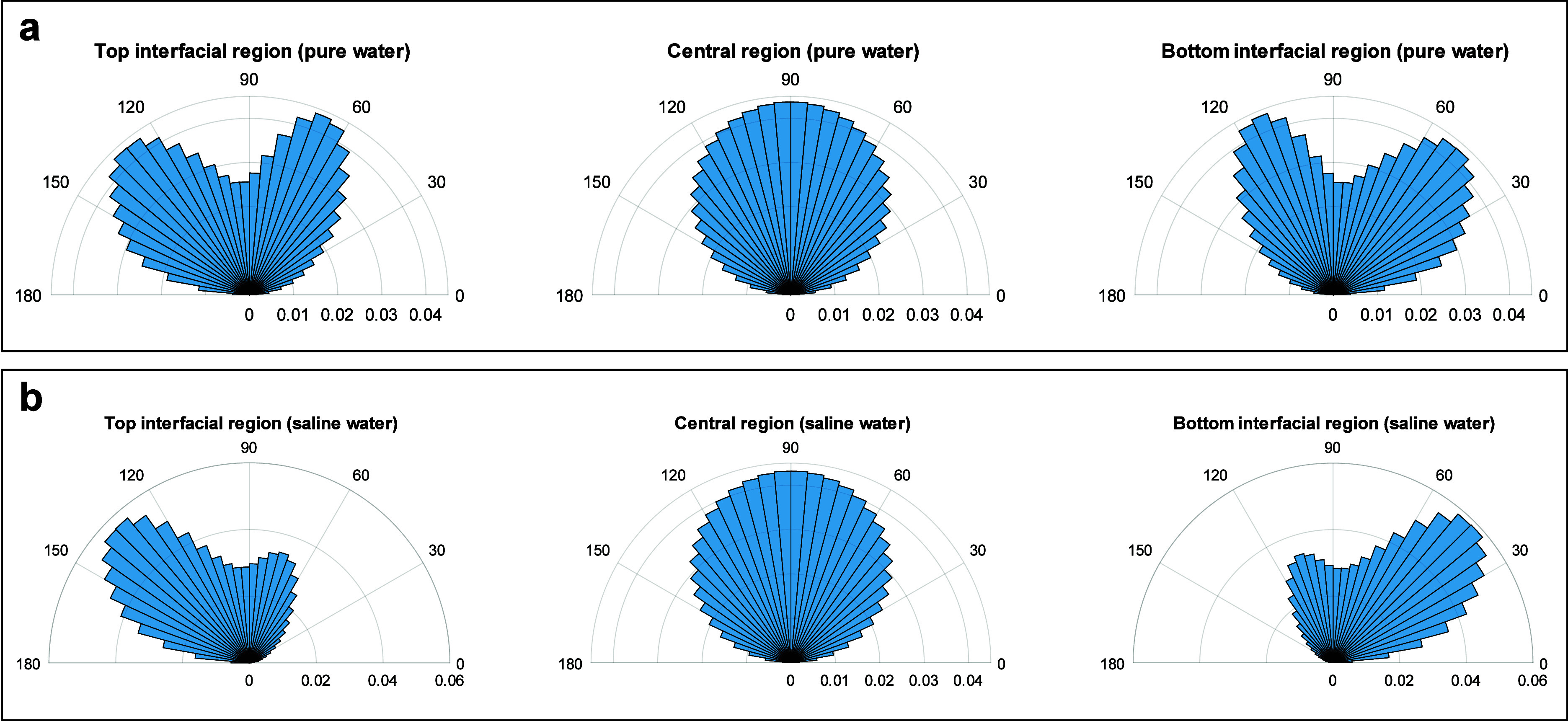
Water molecules’ dipole orientation inside calcite nanochannels. (a) Polar histograms depict the angle between each water molecule’s dipole moment and the *z*-axis (perpendicular to the channel wall) for pure water confined within a 3 nm channel. The channel is segmented into three regions: bottom interfacial (0 < *z* < 0.5 nm), central (0.5 < *z* < 2.5 nm), and top interfacial (2.5 < *z* < 3 nm). In the central region, the majority of water molecules align parallel to the surfaces. Conversely, peaks at θ ≈ 70° and 130° observed in the top interfacial region suggest preferred orientations for dipole moments induced by the surface. (b) Polar histograms representing the orientation distribution of water molecules’ dipole moments under similar conditions as in (a) for saline water (*c* = 2 M). Consistently, no significant change in the trend is observed with salinity, indicating that the orientation of water molecules remains largely unaffected by the presence of salt ions.

We analyzed water molecules’ dipole orientation within a 3 nm channel. As schematically illustrated in Figure 7a, the angle (θ) is defined as the angle between the dipole moment vector (μ) of water molecules and the *z*-axis (perpendicular to the surfaces). Based on the perpendicular permittivity (Figure 3), parallel permittivity (Figure 6), atomic charge, and polarization density (Figure 5) variations inside the channel, we observe that water behaves significantly differently near the surface. These interfacial effects seem to extend up to a distance of 5 Å from the surface. Therefore, for clarity, the channel is segmented into three distinct regions: the bottom interfacial region (0 < *z* < 0.5 nm), the central region (0.5 < *z* < 2.5 nm), and the top interfacial region (2.5 < *z* < 3 nm), as illustrated in Figure 7b. The results are depicted in Figure 8, where polar histograms of dipole angle (θ) are shown for both pure water (Figure 8a) and saline water with a concentration of 2 M (Figure 8b). According to our definition, θ = 0° corresponds to the dipole moment vector being perpendicular to the calcite surface, while θ = 90° implies orientations parallel to the confining surfaces. In the central region, for both pure and saline water cases, we observe an identical orientation distribution with a peak at 90°. This angular distribution implies that the majority of the water molecules are oriented parallel to the confining surfaces regardless of salinity. This aligns with other observations of confined water between graphene and graphene oxide slit nanopores.^23,36^ In the case of the interfacial regions, due to system symmetry, the histograms of the top and bottom interfacial regions mirror each other. In the top interfacial region of pure water, we observe two peaks around 70° and 130°, indicating the initial preorientation of water molecules induced by the calcite surface. With the addition of salt ions to the system, we still observe these peaks, although the peak around 70° decreases. This suggests that while salinity introduces perturbations to the system and causes slight deviations in the orientation distribution of water molecules, the initial preorientation of water molecules induced by the surface remains largely preserved. We found little evidence to support the induced randomness in water orientation proposed by Jalali et al.^35^ as a result of adding salt ions to the solution. We observe the same behavior in the 1 nm channel as well (Figures S2 and S3). In conclusion, the nearly identical patterns of orientation histograms of water molecules in the presence and absence of salt ions suggest that, similar to perpendicular permittivity, parallel permittivity is also controlled by the surface confining effects rather than by salinity effects.

## 4 Conclusion

In this study, molecular dynamics simulations were utilized to quantify the dielectric permittivity of nanoconfined saline fluids. Specifically, calcite was selected as the confining mineral, while NaCl served as the salt, mirroring the composition of geofluids confined within nanoporous geological structures. When water is confined within nanoscale pores it exhibits markedly low perpendicular and high parallel permittivity components. Here, we quantified how salt concentration impacts this anomalous dielectric behavior of nanoconfined water. Our findings indicate that the presence of salt ions only marginally affects both perpendicular and parallel permittivity. Salinity introduces minor perturbations in the atomic charge density, perpendicular polarization density, and the orientation of water molecules’ dipole moments inside the nanochannel. However, it appears that the confinement effects induced by the surfaces are dominant and control the fluids dielectric response rather than salinity effects. These findings imply that computed dielectric permittivities of pure water confined between mineral nanopores at various pressure–temperature conditions can be used to model a broad range of geofluids, i.e., water-salt mixtures. Incorporating these dielectric properties into current geochemical models could improve predictions of geochemical processes in nanoporous environments, including geochemical reaction rates, ion diffusion, and the mobility of dissolved substances within nanoporous rocks and sediments. These processes, in turn, control weathering, mineral dissolution and precipitation, as well as the release of nutrients and contaminants into groundwater.

## Data Availability

The simulation platform used to generate the molecular dynamics results can be accessed through the following link (http://lammps.sandia.gov). Input parameters are available in a data publication that can be accessed through the Utrecht University YODA Portal: https://public.yoda.uu.nl/geo/UU01/S0MHDM.html. Code availability: The authors declare that all necessary data supporting the findings of this study are available within the article and a data publication that can be accessed through the Utrecht University YODA Portal: https://public.yoda.uu.nl/geo/UU01/S0MHDM.html.

## Supporting Information Available

Density functional theory methods; additional details on the convergence of perpendicular permittivity; and the orientation of water molecule dipoles inside the 1 nm calcite channel (PDF)
