# Supplementary material for: The Effect of Salinity on the Dielectric Permittivity of Nanoconfined Geofluids
Source: ACS Earth Space Chem. 2024 Nov 5;8(11):2284–93. doi: 10.1021/acsearthspacechem.4c00210 (PMC11587090; doi:10.1021/acsearthspacechem.4c00210)
Supplement: Supplementary file 1 — sp4c00210_si_001.pdf [file sp4c00210_si_001.pdf]

Supporting information

The effect of salinity on the dielectric permittivity of nanoconfined geofluids

Alireza Chogani*, Helen E. King, Aleksandar Živković, Oliver Plümper
Department of Earth Sciences, Utrecht University, 3584 CB Utrecht, The Netherlands
*Corresponding Author/E-mail: a.chogani@uu.nl

Contents of this file

- Supplementary material 1: Density functional theory (DFT) (page S2)
- Supplementary material 2: $\varepsilon_{\perp}$ convergence (page S3)
- Supplementary material 3: Dipole orientation inside the 1-nm channel (page S4)
- References (page S5)

List of figures

- Fig. S1. Average $\varepsilon_{\perp}$ as a function of time for one of five simulations with conditions H = 3 nm and c = 2 M (page S3)
- Fig. S2. Water molecules' dipole orientation in the bottom half of a 1-nm channel for pure and saline (c = 2 M) water (page S4)
- Fig. S3. Dipole orientation of water molecules in the first and second dense layers near the calcite surface (H = 1 nm) for pure and saline (c = 2 M) water (page S4)

Supplementary material 1: Density functional theory (DFT)

The calcite surface (10-14) was initially relaxed using DFT for further molecular dynamics (MD) simulations. Given that DFT is a quantum-mechanical based method, it allowed us to obtain a very accurate starting geometry for later water molecule addition. As surfaces are known to play a major role in a range of interfacial phenomena (adsorption, non-zero dipole, local defects, etc.), we have used DFT to avoid spurious effects which are hard to be traced in the later stages. DFT calculations were performed using a setup validated by King *et al.*[1]. DFT calculations were performed using a linear combination of atomic orbitals (LCAO) basis set as implemented in the all-electron code CRYSTAL (2017 release)[2,3] where the atoms were described using basis sets from Valenzano *et al.*[4]. The global hybrid B3LYP exchange-correlation functional[5,6] was used without further modifications. The mixing of non-local Fock and semi-local exchange provides a reliable representation of electronic and structural properties of a range of oxide compounds[7-9]. Long-range dispersion corrections were included using the semiempirical D3 approach of Grimme *et al.* with Becke-Johnson damping[10-12]. Bulk calcite was described in the R-3c (#167)[13] space group, as obtained from the American Mineralogist Crystal Structures Database[14]. The Coulomb and exchange series are summed directly and truncated using overlap criteria with thresholds of 10^{-8}, 10^{-8}, 10^{-8}, 10^{-8}, and 10^{-16} as described previously[2,15]. The condition for the self-consistent field (SCF) convergence was set to 10^{-8} au on the total energy difference per cell between two subsequent cycles. Reciprocal space was sampled according to a regular sublattice with a shrinking factor (input IS) of 7. Within DFT, the surface was modelled as a two-dimensional periodic slab, where no three-dimensional periodicity was imposed. To characterize the surface, the surface energy (γ), indicative of thermodynamic stability, is calculated using the following equation:

$$\gamma = \frac{E(n) - n\,E_{bulk}}{2A} \qquad \text{(S1)}$$

where $E(n)$ is the energy of the slab containing n-layers, E_{bulk} the energy of the bulk, and A the area of the slab. The calculated surface energy 0.49 J/m^2 for the calcite (10-14) surface, corresponds well to earlier calculated values[16]. This surface geometry has been utilized as a starting point for subsequent molecular dynamics simulations.

Supplementary material 2: $\varepsilon_\perp$ convergence

In one of our five simulation runs for the 3-nm channel and the highest investigated salt concentration (c = 2 M), the system had not fully converged by 40 ns, prompting us to extend the simulation to 70 ns. The convergence of $\varepsilon_\perp$ over time is shown in Fig. S1. Interestingly, the permittivity values between 40 ns and 70 ns differed by only 1.4%, indicating that the values at 40 ns were already very close to the final stable state. All other simulations were fully converged by 40 ns, so no further extension was needed (as illustrated in Fig. 2 of the manuscript).

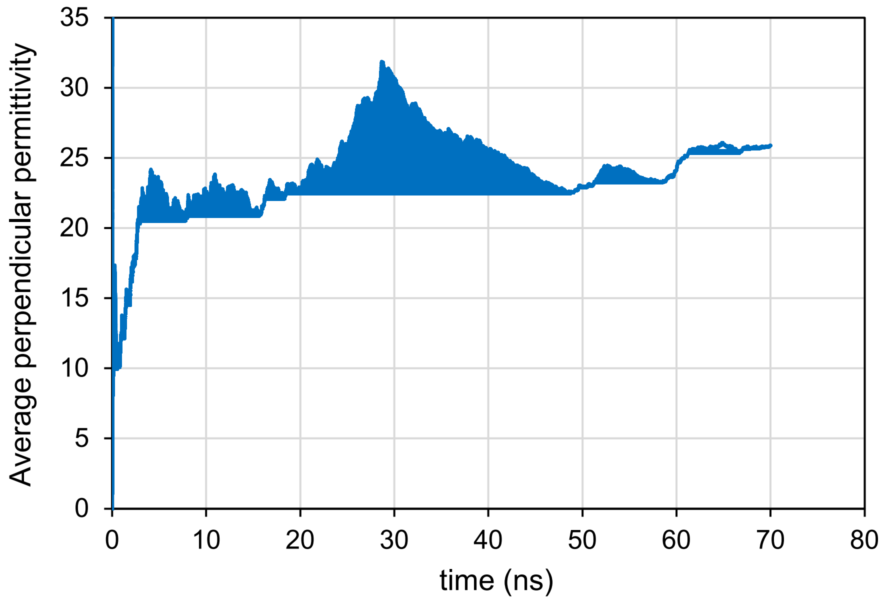

Figure S1. Average $\varepsilon_\perp$ as a function of time for one of five simulations with conditions H = 3 nm and c = 2 M. We extended the simulation to 70 ns to ensure full convergence of the perpendicular dielectric permittivity.

Supplementary material 3: Dipole orientation inside the 1-nm channel

We analyzed the dipole orientation of water molecules within a 1-nm channel for pure and saline (c = 2 M) water. No meaningful differences were observed due to salinity (Fig. S2). As evident in Fig. 4c of the manuscript, water forms two dense layers near the calcite surface. We examined the dipole orientation in these dense layers for both pure and saline water. The orientation distributions for pure and saline water are extremely similar (Fig. S3). Dipole orientations in the first and second interfacial layers are consistent with the findings reported by Söngen *et al.*[17].

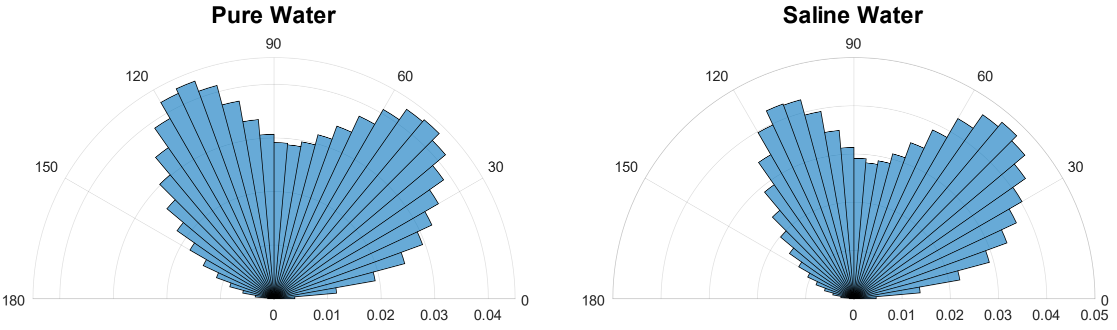

Figure S2. Water molecules' dipole orientation in the bottom half of a 1-nm channel for pure and saline (c = 2 M) water. In both cases, the surface controls dipole orientations, and no significant changes are observed due to salinity.

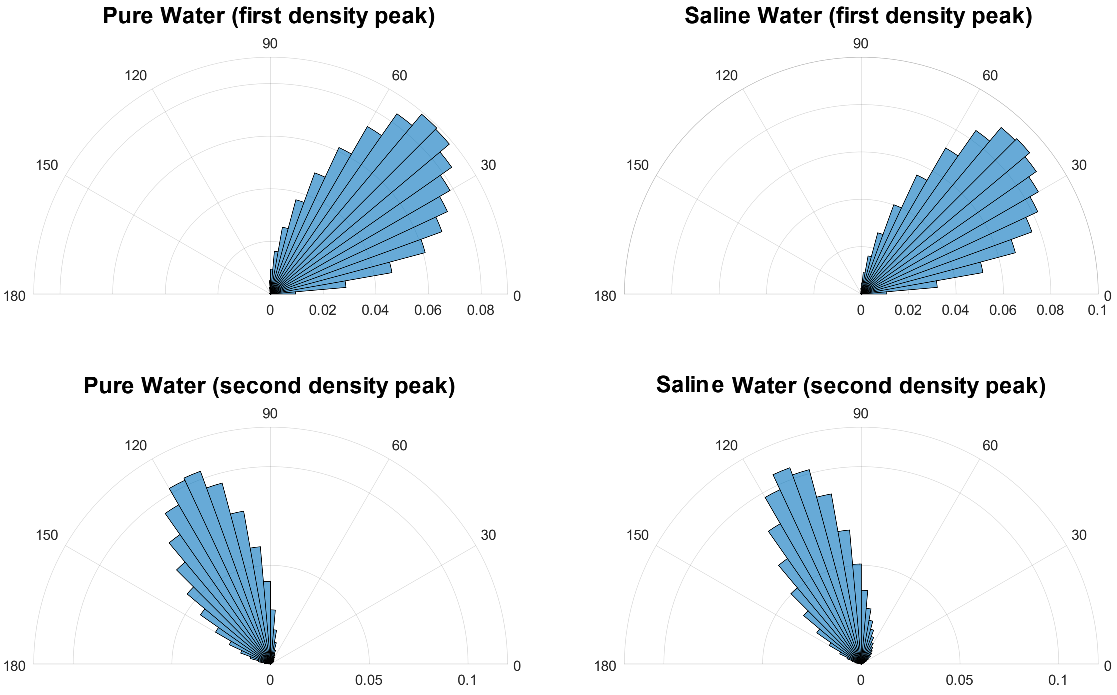

Figure S3. Dipole orientation of water molecules in the first and second dense layers near the calcite surface (H = 1 nm) for pure and saline (c = 2 M) water. No significant changes are observed due to the presence of salt ions in the solution.

References

(1) King, H. E.; Živković, A.; de Leeuw, N. H. Evaluating the effect of ^{18}O incorporation on the vibrational spectra of vaterite and calcite. *Crystals* **2022**, *13*, 48.

(2) Dovesi, R.; Saunders, V. R.; Roetti, C.; Orlando, R.; Zicovich-Wilson, C. M.; Pascale, F.; Civalleri, B.; Doll, K.; Harrison, N. M.; Bush, I. J. CRYSTAL17 user's manual. **2017**.

(3) Dovesi, R.; Erba, A.; Orlando, R.; Zicovich-Wilson, C.; Civalleri, B.; Maschio, L.; Rérat, M.; Casassa, S.; Baima, J.; Salustro, S.; Kirtman, B. Quantum-mechanical condensed matter simulations with CRYSTAL. *WIREs Comput. Mol. Sci.* **2018**, *8*, e1360.

(4) Valenzano, L.; Torres, F. J.; Doll, K.; Pascale, F.; Zicovich-Wilson, C.; Dovesi, R. Ab initio study of the vibrational spectrum and related properties of crystalline compounds; the case of $CaCO_3$ calcite. *Zeitschrift für Physikalische Chemie* **2006**, *220*, 893–912.

(5) Becke, A. D. A new mixing of Hartree–Fock and local density-functional theories. *J. Chem. Phys.* **1993**, *98*, 1372–1377.

(6) Lee, C.; Yang, W.; Parr, R. G. Development of the Colle-Salvetti correlation-energy formula into a functional of the electron density. *Phys. Rev. B* **1988**, *37*, 785–789.

(7) Muscat, J.; Wander, A.; Harrison, N. M. On the prediction of band gaps from hybrid functional theory. *Chem. Phys. Lett.* **2001**, *342*, 397–401.

(8) Harrison, N. M. First principles simulation of surfaces and interfaces. *Comput. Phys. Commun.* **2001**, *137*, 59–73.

(9) Patel, M.; Mallia, G.; Liborio, L.; Harrison, N. M. Water adsorption on rutile TiO_2(110) for applications in solar hydrogen production: A systematic hybrid-exchange density functional study. *Phys. Rev. B* **2012**, *86*, 045302.

(10) Grimme, S.; Antony, J.; Ehrlich, S.; Krieg, H. A consistent and accurate ab initio parametrization of density functional dispersion correction (DFT-D) for the 94 elements H-Pu. *J. Chem. Phys.* **2010**, *132*, 154104.

(11) Grimme, S.; Ehrlich, S.; Goerigk, L. Effect of the damping function in dispersion corrected density functional theory. *J. Comput. Chem.* **2011**, *32*, 1456–1465.

(12) Grimme, S.; Hansen, A.; Brandenburg, J. G.; Bannwarth, C. Dispersion-Corrected Mean-Field Electronic Structure Methods. *Chem. Rev.* **2016**, *116*, 5105–5154.

(13) Graf, D. L. Crystallographic tables for the rhombohedral carbonates. *Am. Mineral.* **1961**, *46*, 1283–1316.

(14) Downs, R. T.; Hall-Wallace, M. The American Mineralogist crystal structure database. *Am. Mineral.* **2003**, *88*, 247–250.

(15) Pisani, C.; Dovesi, R.; Roetti, C. *Hartree-Fock ab initio treatment of crystalline systems;* Springer Science & Business Media: 2012; Vol. 48.

(16) Elbashier, E.; Hussein, I.; Carchini, G.; Pour, A. S.; Berdiyorov, G. R. Effect of strain on gas adsorption in tight gas carbonates: A DFT study. *Comp. Mater. Sci.* **2021***, 188*, 110186.

(17) Söngen, H.; Schlegel, S. J.; Morais Jaques, Y.; Tracey, J.; Hosseinpour, S.; Hwang, D.; Bechstein, R.; Bonn, M.; Foster, A. S.; Kühnle, A. Water orientation at the calcite-water interface. *J. Phys. Chem. Lett.* **2021***, 12*, 7605–7611.
